# New equipment optimized for emulsified vaccine prepation in the hospital

**DOI:** 10.1186/2051-1426-1-S1-P196

**Published:** 2013-11-07

**Authors:** Stephane Ascarateil, Marie Eve Koziol

**Affiliations:** 1 Biologicals and Injectables, SEPPIC, Puteaux, France; 2 Biologicals and Injectables, SEPPIC Inc, Fairfield, NJ, USA

## 

Developing stable formulations is generally a common target to obtain efficacious emulsified vaccines. Process used to perform water in oil (W/O) emulsions are not all equivalent regarding vaccines physico-chemical parameters. Equipment generally used to realise emulsified vaccines can be high shear mixers, vortex, syringes, or syringes connectors. It was already reported that applied process has a direct impact on quality, stability and particles size of dispersed phase (Ascarateil, Isbtc 2008). Moreover it can influence directly biological effects of the vaccine, and especially its action against tumor (Koh, 2006). Differences of process from a clinical trial to another can be a root of misunderstanding on the results obtained, and certainly the cause of unexpected variations. In the hospital obtaining reproducible and stable W/O emulsions is a challenge for the pharmacy or the operators involved. It is important to ensure sterility while vaccine is prepared at patient’s bed. The equipment used for this preparation has thus to be disposable, developed in order to lower human errors to the max, and be not time consuming. Here we present the development of a new equipment optimized for automatic W/O emulsion process. It is a closed system allowing to maintain sterility while emulsification process is running. Process has been optimized according to already existing experiences on this kind of process. The machine is able to repeat low and fast rhythm cycle as they are performed in the hand I connector process and the operation is blocked after a determined number of cycles. Emulsions obtained were analysed for their viscosity, microscopic appearance and their particle sizes with Mastersizer™ equipment. Placebo emulsions have to present an average particle size of 1µm in order to validate the process parameters. Emulsions were put in a stability program for one month a three different temperatures with the aim that particle sizes should vary during this period. The emulsifying adjuvants used for the qualification of this new equipment were Montanide™ ISA 51 and Montanide™ ISA 720.

